# Assessing Heterogeneity of Osteolytic Lesions in Multiple Myeloma by ^1^H HR-MAS NMR Metabolomics

**DOI:** 10.3390/ijms17111814

**Published:** 2016-10-31

**Authors:** Laurette Tavel, Francesca Fontana, Josè Manuel Garcia Manteiga, Silvia Mari, Elisabetta Mariani, Enrico Caneva, Roberto Sitia, Francesco Camnasio, Magda Marcatti, Simone Cenci, Giovanna Musco

**Affiliations:** 1 Biomolecular NMR Unit, Division of Genetics and Cell Biology, San Raffaele Scientific Institute, 20132 Milano, Italy; tavelfuchs.laurette@hsr.it (L.T.); silvia@mestrelab.com (S.M.); 2 Protein Transport and Secretion Unit, Division of Genetics and Cell Biology, San Raffaele Scientific Institute, 20132 Milano, Italy; francesca.fontana@wustl.edu (F.F.); garciamanteiga.josemanuel@hsr.it (J.M.G.M.); sitia.roberto@hsr.it (R.S.); 3 Università Vita-Salute San Raffaele, 20132 Milano, Italy; 4 Age Related Diseases Unit, Division of Genetics and Cell Biology, San Raffaele Scientific Institute, 20132 Milano, Italy; mariani.elisabetta@hsr.it; 5 Centro Interdipartimentale Grandi Apparecchiature, Università di Milano, 20132 Milano, Italy; enrico.caneva@unimi.it; 6 Department of Orthopaedics and Traumatology, Orthopaedics, San Raffaele Scientific Institute, 20132 Milano, Italy; camnasio.francesco@hsr.it; 7 Department of Oncohematology, Hematology and Bone Marrow Transplantation Unit, San Raffaele Scientific Institute, 20132 Milano, Italy; marcatti.magda@hsr.it

**Keywords:** high-resolution magic-angle spinning (HR-MAS), metabolomics, multiple myeloma, nuclear magnetic resonance (NMR), osteolysis

## Abstract

Multiple myeloma (MM) is a malignancy of plasma cells characterized by multifocal osteolytic bone lesions. Macroscopic and genetic heterogeneity has been documented within MM lesions. Understanding the bases of such heterogeneity may unveil relevant features of MM pathobiology. To this aim, we deployed unbiased ^1^H high-resolution magic-angle spinning (HR-MAS) nuclear magnetic resonance (NMR) metabolomics to analyze multiple biopsy specimens of osteolytic lesions from one case of pathological fracture caused by MM. Multivariate analyses on normalized metabolite peak integrals allowed clusterization of samples in accordance with a posteriori histological findings. We investigated the relationship between morphological and NMR features by merging morphological data and metabolite profiling into a single correlation matrix. Data-merging addressed tissue heterogeneity, and greatly facilitated the mapping of lesions and nearby healthy tissues. Our proof-of-principle study reveals integrated metabolomics and histomorphology as a promising approach for the targeted study of osteolytic lesions.

## 1. Introduction

Cancer-induced bone disease is one of the commonest and most severe complications in cancer, leading to severe pain, compression, and fractures, with a profound impact on quality of life, morbidity, and survival. Frequently attracted to skeletal sites, cancer cells establish vicious interactions with the bone microenvironment, occupying functional niches and exploiting local homeostatic factors to gain fitness, drug resistance, and growth support. Multiple myeloma (MM) is a cancer of bone marrow resident plasma cells, which grow abnormally at multiple skeletal sites and subvert physiologic bone remodeling by suppressing osteoblastic bone formation and promoting osteoclastic bone resorption, thereby inducing systemic bone loss and multifocal bone destruction [1,2].

Skeletal cancer lesions experience heterogeneous metabolic conditions owing to different vascularization, pH, and mechanical strain. From a clinical standpoint, the evaluation of individual lesions is important to prevent severe complications such as skeletal-related events. Recent evidence demonstrated genetic heterogeneity within the same tumor lesion, which may explain tumor progression and resistance to treatment [3]. Understanding such heterogeneity may help understand the pathobiology of cancer-induced bone disease in order to develop novel therapeutic targets.

Metabolomics, the comprehensive assessment of small metabolites in a biological system, has been shown to provide relevant insights in complex diseases, with many potential clinical applications [4,5,6]. In cancer, metabolic profiling may help decipher the vicious interactions established by MM cells with the bone environment, possibly amenable to therapeutic manipulation. High-resolution magic-angle spinning (HR-MAS) nuclear magnetic resonance (NMR) spectroscopy is becoming a routine technique for investigating metabolic profiles from ex vivo tissue samples [7,8,9]. This analytical method takes advantage of fast acquisition times, minimal sample preparation, and high reproducibility. Moreover, being spared from destruction, samples analyzed by HR-MAS are available for further analyses, such as histology, proteomics, or gene expression, thus allowing for direct comparison of data obtained with different techniques on the same sample [6,10,11,12]. The possibility to correlate metabolic alterations and histopathologic features of a surgical tumor specimen may help decipher tumor heterogeneity and gain better molecular insights into tumor pathogenic mechanisms [5,13]. Indeed, ^1^H HR-MAS NMR is now increasingly used for the characterization of tumor lesions in humans, including brain [14,15], breast [16], liver [17], colon [18], and prostate cancers [19]. Notably, ^1^H HR-MAS NMR has also been applied to the study of articular cartilage degradation [20], intervertebral disc degeneration [21], gamma-radiated bones of non-cancer mouse models [22], and human bone mineral tissue [23]. However, to the best of our knowledge, ^1^H HR-MAS NMR has never been applied to achieve metabolic analysis of bone tumor lesions in human patients.

Here, we carried out a proof-of-concept study of the feasibility of ^1^H HR-MAS NMR to address local heterogeneity in cancer-induced osteolysis. To this aim, we analyzed multiple biopsy specimens from one case of pathological fracture caused by MM. Two macroscopically different types of tissue were identified within a single specimen of approximately 3 cm diameter, and adjacent muscle was used as a third region for comparison. Multivariate analyses on normalized metabolite peak integrals allowed to identify intergroup differences and to cluster samples in accordance with a posteriori histological findings. To further investigate the relationship between histological and NMR features, morphological data and metabolite profiling were compared independently of group assignment. Collectively, our results show that NMR holds potential to complement histological analysis in the targeted study of cancer osteolytic lesions.

## 2. Results

### 2.1. ^1^H HR-MAS Spectra Highlight Differences among Multiple Biopsy Specimens

**Figure 1:**
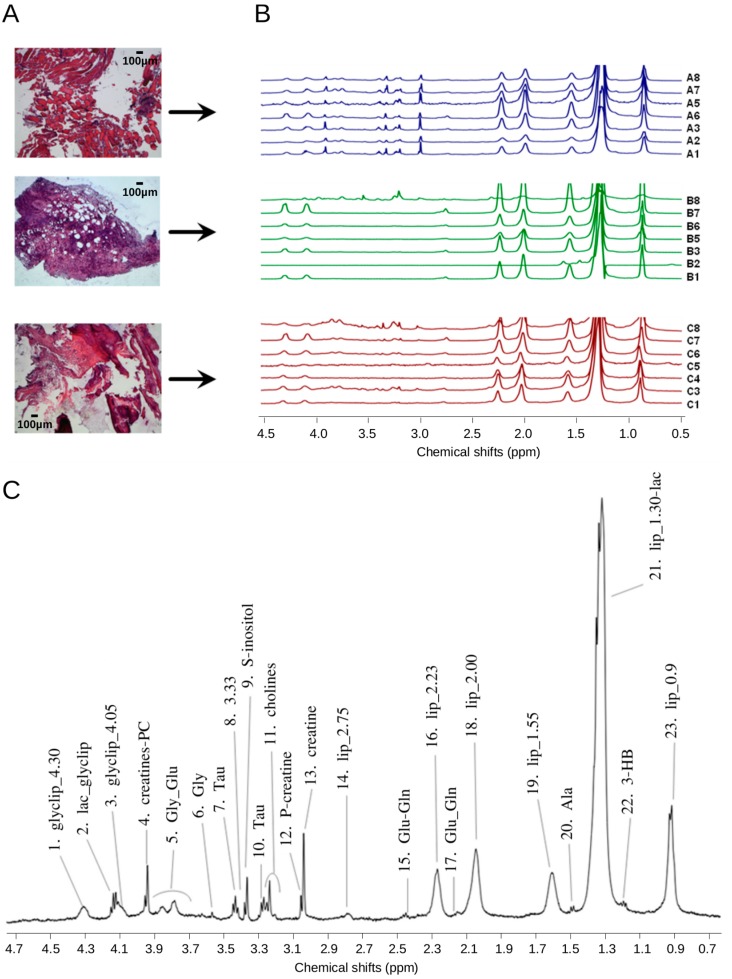
^1^H HR-MAS (high-resolution magic-angle spinning) spectra of multiple biopsy specimens. (**A**) From top to bottom: representative hematoxylin and eosin-stained morphological sections of samples belonging to group A, B, and C, respectively; (**B**) Overlay of the spectra acquired on the different specimens; the labels on the right indicate group and sample ID; (**C**) Representative ^1^H 1D-noesygppr HR-MAS nuclear magnetic resonance (NMR) spectrum of group A sample. Assignment was made on the basis of literature references [7,22,24,25,26,27]. Peaks were calibrated using DSS (4,4-dimethyl-4-silapentane-1-sulfonic acid) peak at 0 ppm as reference. Numbers identify binning regions as listed in Supplementary Table S1. (1) glycerolipids; (2) lactate, glycerolipids; (3) glycerolipids; (4) creatine and phosphocreatine; (5) glycogen, glucose; (6) glycine; (7) taurine; (8) unknown; (9) scyllo-inositol; (10) taurine; (11) choline, *O*-phosphocholine, and glycerophosphocholine; (12) phosphocreatine; (13) creatine; (14) triglyceride =CH–CH_2_–CH=; (15) glutamate and glutamine; (16) triglyceride CH_2_–CH_2_CO; (17) glutamate and glutamine; (18) triglyceride CH=CH–CH_2_; (19) triglyceride CH_2_–CH_2_CO; (20) alanine; (21) triglyceride (CH_2_)n, lactate and threonine; (22) 3-hydroxybutyrate; (23) triglyceride CH_3_–(CH_2_)n.

^1^H HR-MAS spectra were obtained for seven muscle (group A: A1–A3, A5–A8), seven oily tumor (group B: B1–B3, B5–B8), and seven calcified tumor (group C: C1, C3–C8) samples (Figure 1A). As control spectrum we used a mixed muscle-tumor specimen (A4/B4). Figure 1B and Supplementary Figure S1A show the superposition of the ^1^H HR-MAS NOESY spectra of the three different sample groups and the corresponding average spectra, respectively. Visual inspection of the spectra highlighted different profiles for the three sample groups in accordance with the classification obtained by macroscopic examination of the tumor sample prior to insert preparation. In particular, spectra deriving from muscle samples contained more peaks as compared to tumor or calcified, consistent with the higher number of metabolites expected for a specimen with high cytoplasmic content (Figure 1A,B, Supplementary Figure S1A). Spectra were assigned based on previously published data [7,24,25,26,27] (Figure 1C, Supplementary Table S1) and the relative amounts were compared between different groups (ANOVA) (Supplementary Table S1). As expected, creatine and phosphocreatine were particularly enriched in muscle samples (*p* < 0.01) as compared to tumor specimens (groups B or C) (Supplementary Figure S2 and Table S1). Also taurine, highly abundant in the cytoplasm, was more abundant in muscle as compared to tumor samples, reflecting its high cytoplasmic content. In contrast, tumor samples had higher content of glycerolipids at 2.00 and 2.23 ppm (*p* < 0.05, Supplementary Figure S2 and Table S1) likely originating either from membrane phospholipids, due to high cancer cellularity, or from triacylglycerides and fatty acid chains composing lipid droplets.

### 2.2. Sample Recovery from HR-MAS Rotors Allows Histology and Histomorphometry Analysis

**Figure 2:**
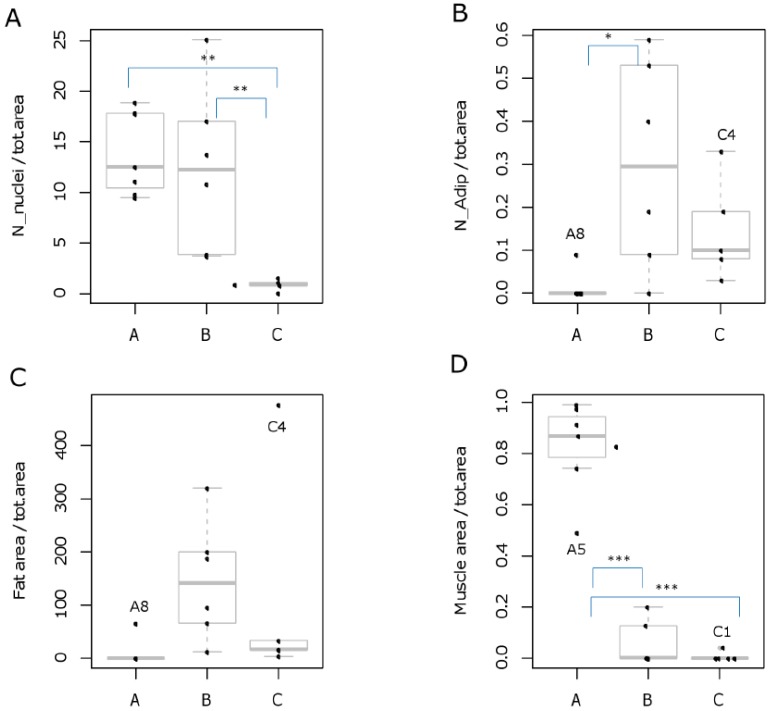
Statistical analysis of histomorphometry of A, B and C samples. Samples were analyzed after ^1^H HR-MAS NMR acquisition to evaluate the number of nuclei (**A**); number of adipocytes (**B**); fat area (**C**); and muscle area (**D**). All data were normalized to total area and box-whisker plots were used to visualize the results. The bars outside the plot represent data outside 1.5 times the IQR (interquartile range), and points outside the bars are outliers. Outlier samples are identified with a label. Statistical significance was evaluated by ANOVA (Tukey’s post-hoc tests, * *p* < 0.05, ** *p* < 0.01, *** *p* < 0.001).

After spectroscopic analysis, we recovered samples for histological analysis. This was feasible for seven muscle specimens (A1–A3, A5–A8), six out of seven oily (B1–B3, B6–B8), and five out of eight calcified tumor (C1, C3, C4, C6, C8) samples (100%, 86%, and 63%, respectively). Histology confirmed all group A samples to be muscle fibers. Most B samples consisted of tightly packed tumor cells and fatty areas. C samples contained eosinophilic extracellular matter compatible with bone residues and connective tissue with reduced cellularity. Histological examination also revealed the erroneous classification of two samples, C8 and B8, which presented a high concentration of small cells, with a muscle fiber trapped in B8 (as shown below). By applying histomorphometry for each section, we counted the number of nuclei (Figure 2A) and adipocytes (Figure 2B) or fat droplets and quantified the areas associated either to fat (Figure 2C) or muscle (Figure 2D). Statistical analysis of these measurements confirmed significant morphological differences among groups (*p* < 0.05 by one-way ANOVA/Tukey’s post-test, Figure 2). Group A showed a high concentration of nuclei (Figure 2A), with each fiber containing on average 200–300 nuclei, arranged on the border of the fiber in non-damaged tissue. Little variability was noted in this group, accounted for by few artifacts, and by the sporadic presence of suffering fibers with centralized nuclei or loose connective tissue substitution. Group B showed intense areas of packed tumor cells with a high number of vessels and with bigger nuclei distributed around fatty areas. These areas of less intense color were immersed in fibrillar tissue and big fat droplets area, with poorly organized structure and irregular disposition of collagen fibers. Of note, in group C we also observed the presence of calcified tissue compatible with bone. In accordance with the calcified nature of the sample, the number of cells and the abundance of connective tissue were reduced as compared to groups A and B, and Havers channels and osteoclasts were detected.

### 2.3. Unsupervised Multivariate Statistical Analysis of ^1^H HR-MAS NMR Data

**Figure 3:**
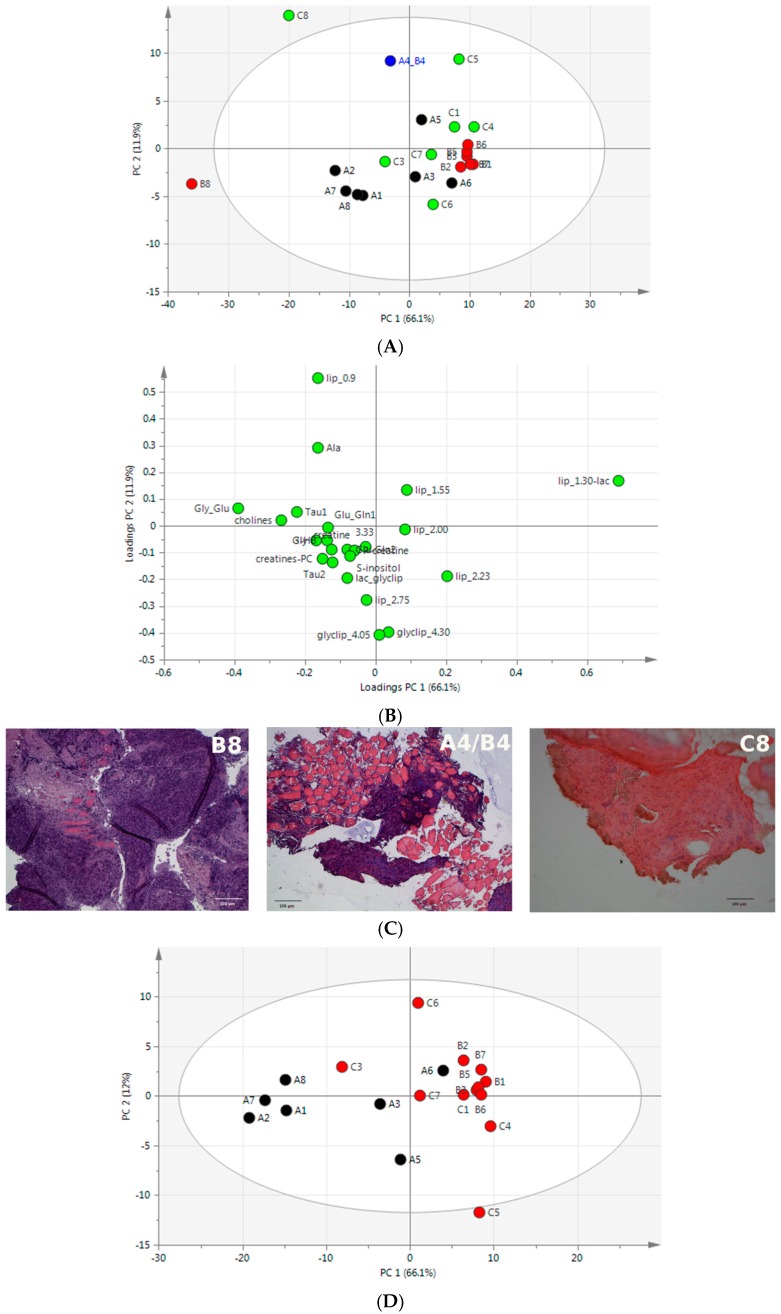

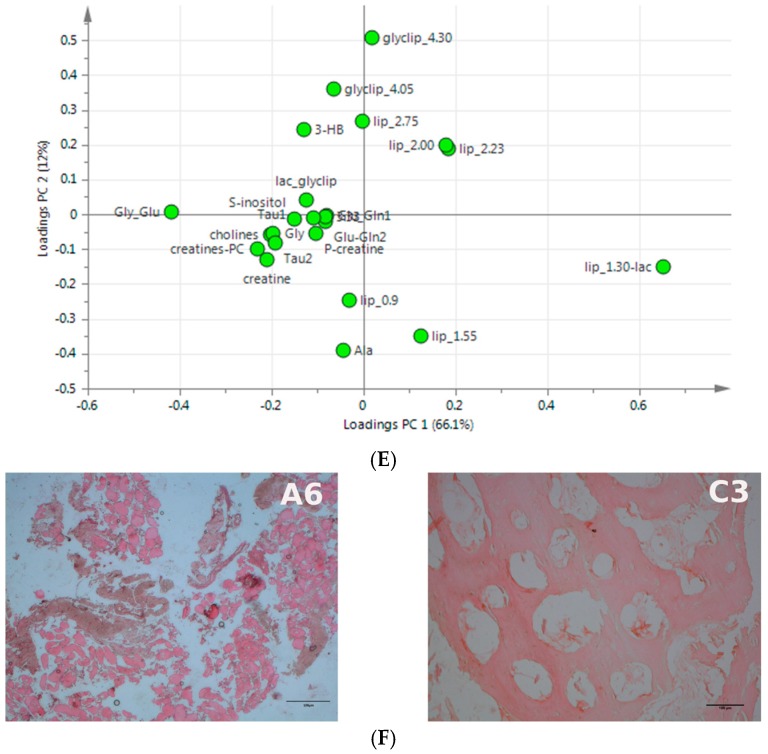
Multivariate analysis of NMR spectra and histological features. NOESY spectra acquired from samples of different origins were statistically analyzed after binning. Classification of specimens was based on their morphological appearance at the time of sampling. (**A**) Score plot showing the first two principal components, with black, red, and green dots, respectively corresponding to muscle (group A), oily tumor (group B), and calcified tumor samples (group C). The quality control sample A4/B4, prepared by mixing muscle and tumor specimens, is colored in blue. The oval represents the 95% confidence interval of the analysis; (**B**) Loadings plot of the analysis performed in (**A**). Profiled bucket metabolites and their ppm values are shown for those regions displaying loadings responsible for separation along the first two principal components (PC1, PC2); (**C**) Histological staining of mixed control samples (A4/B4) and of outliers (B8, C8) with hematoxylin and eosin (5X, Axioplan Leica). Upon staining of 5 μm sections, nuclei appear stained by hematoxylin (violet/blue), muscle fibers by eosin (red), loose connective tissues are weakly stained, and fat remains white. Scale bar = 100 µm; (**D**) PCA score plot comparing muscle samples (group A, in black) with tumor samples (groups B and C, in red) after exclusion of the two outliers (B8, C8) and the mixed control sample (A4/B4); (**E**) Loadings plot of the analysis performed in (**D**); (**F**) Histological staining of misclassified samples (A6, C3). Sample A6 shows tumor cells between muscle fibers. Sample C3 shows more organized calcified tissue. Scale bar = 100 µm.

In order to identify differences and similarities between sample groups and to obtain an unbiased overview of the NMR dataset we applied principal component analysis (PCA). The PCA score plot and loadings plot of NOESY data are shown in Figure 3A,B, respectively. The first two principal components accounted for 78% of variance, showing that separation of samples based on their type of origin was mainly achieved through the first principal component (PC1), which had a large positive loading for bin 21 (around 1.3 ppm) and negative loading for glycogen–glucose (bin 5, around 3.7 ppm) (Supplementary Table S1). The quality control sample A4/B4, prepared mixing muscle and tumor specimens, contained both tumor cells and muscle fibers. As expected, in agreement with its mixed origin, its PCA score was within 95% confidence, even though it was far from the centroids of both groups A and B. Notably, the PCA score plot was characterized by the presence of two outliers (B8 and C8 samples), which were outside the 95% confidence interval of the dataset. These samples, during insert preparation for ^1^H HR-MAS experiments, were classified by visual inspection as oily and calcified tumors, respectively. However, histological examination revealed an erroneous classification, as assessed by the presence of high concentration of small cells in both C8 and B8 samples, with a muscle fiber trapped in B8 (Figure 3C).

Based on these findings, we repeated PCA on the NMR dataset excluding the misclassified samples (B8 and C8). The PCA score plot showed a clear separation between muscle and tumor samples, still highlighting the presence of two group outliers (i.e., C3 and A6 (Figure 3D)). Interestingly, histological analysis of sample A6 revealed the presence of tumor cells in between muscle fibers (Figure 3F), whereas sample C3 appeared to be highly calcified and to contain more organized calcified tissue as compared to the other calcified samples (Figure 3F).

### 2.4. Integrated Analysis of Histomorphometric Features and NMR Metabolic Profiles

**Figure 4:**
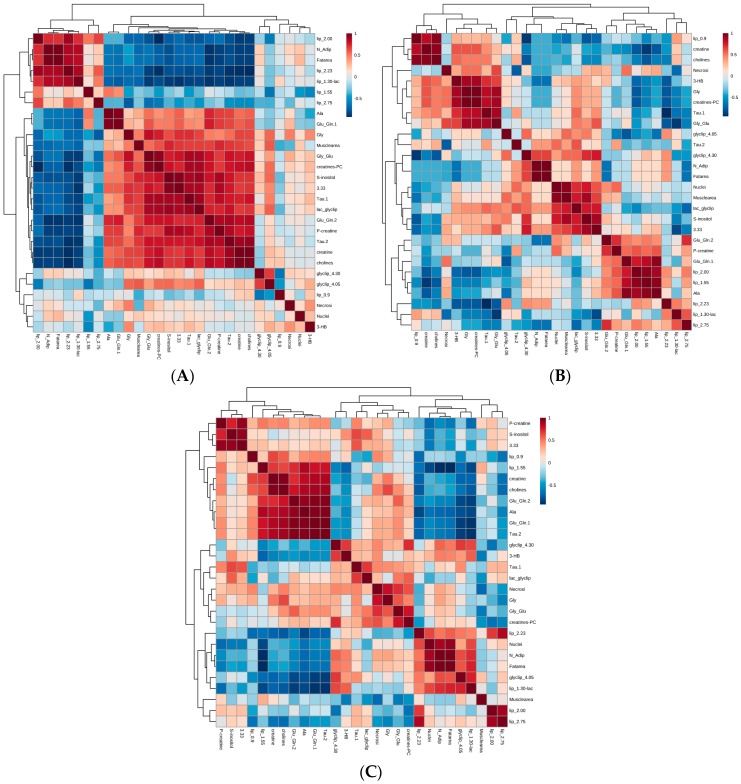
Integrated correlation analysis of histomorphometry and ^1^H HR-MAS NMR profiling data. Joint correlation heatmaps consisting of histomorphometry and ^1^H HR-MAS NMR profiling datasets (including 5 histomorphometric features and 23 NMR bins/metabolites) obtained considering (**A**) all samples without outliers (*n* = 13); (**B**) only muscle samples (*n* = 6); (**C**) only tumor samples (including both B and C samples, *n* = 7). In each matrix histomorphometry data were normalized to total area and scaled to range, while NMR data were normalized to the sum of intensities and Pareto-scaled. NMR bins/metabolites are consistent with Supplementary Table S1. The color of each cell depicts the Spearman-rank correlation coefficients and clusters according to Euclidean distances (*pheatmap* library in R) using a thermal scale from anticorrelation (*r* = −1, blue) through uncorrelated (*r* = 0, white) to correlated (*r* = 1, red). Histomorphometry and NMR data are explicitly labeled on the bottom and on the right of the symmetric matrix, respectively. On the top and on the left of the matrices, dendrograms of the clustering are shown.

It has been recently shown that the analysis of individual metabolite variations associated with a histological approach may better define the complexity of pathologic samples and disease biology [28,29]. We therefore asked whether we could systematically address the relationship between morphological and NMR features. We have shown in the previous paragraph that it is possible to associate typical morphological characteristics to different metabolic signatures. In line with this, samples classified as outliers based on metabolic features had been misclassified by preliminary visual inspection, as subsequently confirmed by histological analysis. To further investigate the relationship between morphological and NMR features, we set out to correlate histomorphological data, normalized spectral peak integrals and metabolite profiling. To this aim, as shown in Figure 4, we created a correlation matrix combining histomorphometric and metabolic data using MetaboAnalyst, an established metabolomics tool-suite. In order to achieve a stringent correlation analysis, the input dataset matrix included only those samples for which both histomorphometry and NMR data were available. Moreover, we excluded those samples judged as outliers or misclassified based on the NMR PCA shown in Figure 3. This process resulted in the analysis of 13 samples in Figure 4A, 6 samples in Figure 4B, and 7 samples in Figure 4C.

As in previous analyses, NMR metabolic data were normalized to the sum of intensities and Pareto-scaled (see Methods for details). The two datasets were then merged, and a correlation matrix was obtained based on Spearman rank-order correlation. Considering the correlation matrix generated using all types of samples (Figure 4A) we observed that typical intracellular and cytosolic metabolites (e.g., taurine, creatine, phosphocreatine, glutamate, glutamine and glycine) correlate with the presence of muscle fibers in the specimen. Conversely, lipidic regions correlate with fat area and number of adipocytes. Similar correlations were observed when only muscle samples were analyzed (Figure 4B). Interestingly, analysis of tumor samples alone highlighted a specific correlation between lipids at 2.75 ppm, 2.23 ppm, and 2.0 ppm, with fat area and adipocytes that were not present in muscle samples alone, suggesting a different lipidic composition in tumor specimens (Figure 4C). Taken together, our data indicate a good concordance between morphological and chemical shift features, which could be exploited to predict tissue composition on the basis of ^1^H HR-MAS spectra, or to increase the specificity of metabolomic analyses on intact tissue.

## 3. Discussion

We have analyzed multiple biopsy specimens from one case of a pathological fracture caused by MM to evaluate the feasibility of an integrated approach combining ^1^H HR-MAS and multivariate analysis with histomorphological information to study osteolytic lesions. To our knowledge, this is the first study using ^1^H HR-MAS NMR to investigate human bone cancer biology, as previous applications of the technique have been reported only on human bone mineral or on bones of non-cancer mouse models [22,23]. The aim of this study was therefore to verify whether ^1^H HR-MAS is applicable to human bone tumor samples, and whether it is able to highlight differences among specimens sampled in morphologically different but anatomically close areas. Multivariate analysis of the ^1^H HR-MAS NMR spectra acquired on the biopsies showed that the main spectral differences between muscle (group A) and tumor tissues (groups B and C) concern the glycogen–glucose region at around 3.7 ppm and the fatty acids methyl groups resonating at 1.3 ppm. The high content of carbohydrates in group A most likely derives from glycogen stored in muscle cells, whereas the fatty acids methyl groups, highly present in the heterogeneous tumor samples, might originate from either phospholipids of membrane-rich tissue with high cellular density, or from triacylglycerides and/or fatty acid chains composing lipid droplets. Notably, subsequent histomorphometric analysis highlighted a high cellular content in districts defined as oily as opposed to calcified ones, but it was not possible to appreciate significant differences in the fat areas of the two tumor sample groups. Accordingly, both univariate (Supplementary Table S1) and multivariate analysis (data not shown) of ^1^H HR-MAS data were not able to discriminate between oily and calcified tumors, yet lipid composition appeared slightly different, as suggested by the peak at 2.75 ppm that was significantly lower in the calcified tumor samples as compared to the oily ones (Supplementary Table S1 and Figure S2). Interestingly, correlation matrices between morphological and NMR features hinted at the possible origin of lipidic differences within tumor specimens (Figure 4C). For example, lipid peaks resonating at 2.75 ppm, 2.23 ppm, and 2.00 ppm were part of a cluster that strongly correlated with the number of adipocytes and fat area. In line with this observation, several spectroscopic studies have highlighted differences in the lipid compositions in tumor cells and tissues, as observed, for example, in malignant human renal tissues or human brain tumor biopsies [30,31]. Our proof-of-principle study of intralesion heterogeneity was not designed to identify metabolites capable of discriminating different tumor areas. Indeed, the lipidic differences observed between the two morphologically distinct areas of the tumor were not significant (see Supplementary Table S1), with the notable exception of lipid 2.75, which suggests the existence of a differential lipid composition in morphologically distinct tumor areas.

In perspective, the use of larger cohorts of heterogeneous bone lesions of different origins could help determine how different lipidic signals corresponding to either phospholipids (cell membrane) or triglycerides (adipocyte-rich fat areas) discriminate different lesion areas or tumor types, providing relevant information on cancer biology.

In highly heterogeneous tissues, such as bone tumors and metastases, interpretation of data deriving from homogenizing or otherwise destructive procedures can be extremely challenging, due to the different cellular populations and local conditions involved. Our work offers proof-of-concept evidence that the histological assessment of bone cancer can be integrated with intact-tissue ^1^H HR-MAS NMR that enables unbiased metabolic analysis by excluding misclassified samples and by correlating tissue composition to specific spectral features. Although our data were collected from a single tumor, our study demonstrates the feasibility of combining HR-MAS and histology studies on patient-derived bone tumor samples. Future work is required to validate this approach for the study of cancer metabolism and response to treatments, in order to gain deeper insight in bone tumor biology. Attesting to clinical translatability, ^1^H HR-MAS, thanks to its execution rapidity and capacity to preserve samples for further analyses, has already shown promising results in other cancers. For example, it has been successfully adopted to determine sample quality in real-time during surgery [32], or to aid diagnosis in tumor biopsies [12,33,34]. In the field of bone cancer, further studies may extend ^1^H HR-MAS analyses to larger patient cohorts to characterize and compare pathologic conditions.

Moreover, the general concordance between metabolic fingerprinting and tissue composition may offer valuable clinical applications, helping to rapidly and efficiently select significant samples for pathological analysis (e.g., to avoid areas of necrosis or reactive tissue). The examination of relatively small amounts of tumor tissues could then be used to assess slight differences in the biochemical profile of the tissue samples prior to histopathology. In this respect, ^1^H HR-MAS NMR spectroscopy is increasingly emerging as a highly versatile tool [6,11,13,14,15,17,18].

In conclusion, herein we have shown that human bone cancer specimens can be analyzed to obtain reproducible ^1^H HR-MAS NMR spectra, and then fixed to produce histological slides spanning the entire sample area. Multivariate analyses on normalized metabolite peak integrals showed samples to cluster in accordance with a posteriori histological findings, and identified intergroup differences. Integrating metabolic profiling and histomorphometry is a promising approach to address heterogeneous bone tumor lesions, establish relevant correlations between metabolic and morphological features, and develop translatable fingerprinting strategies.

Overall, our work provides proof-of-principle evidence in support of a novel, unbiased, rapid, and inexpensive approach that may help the assessment of skeletal areas of cancer-induced osteolysis, providing information of potential biomedical, prognostic and therapeutic value.

## 4. Materials and Methods

### 4.1. Case History and Sampling

A 68-year-old female, with no previous history of malignancy, was admitted to the San Raffaele Orthopaedics Unit for pathological fracture of the left femur of undetermined origin. Conventional radiographies showed a displaced subtrochanteric fracture, with multiple fragments and possible extra-osseous involvement, and diffuse bone loss. A 99Tc-MDP (technetium-99 conjugated with methylene diphosphonate) bone scan showed only little accumulation at the site of fracture, and lack of tracer accumulation at distant sites suspect for bone lesions. Creatinine and calcium levels were normal (0.93 mg/dL and 1.83 mmol/L), and the finding of anemia was partially explained by the presence of trauma. Upon serum protein electrophoresis (SPEP) a monoclonal peak was identified and quantified over 35 g/L. The later-confirmed diagnosis was IgGK myeloma with 46% of bone marrow clonal plasma cells and multiple bone lesions. Because of the pathological fracture, the patient underwent resection and prosthetic replacement of the proximal femur, with good functional results on mobility and reduction of pain. During surgical procedure, biopsy specimens were collected from the osteolytic lesion and the surrounding muscle, immediately snap-frozen in liquid nitrogen, and stored at −80 °C. Sampling was performed during the resection phase, with no requirement for additional procedures, nor prolongation of surgery. Informed consent was obtained in accordance with the declaration of Helsinki. Approval for primary sample use was obtained from the Institutional Review Board of the San Raffaele Scientific Institute.

### 4.2. Sample Preparation for NMR

One bulk tumor sample of approximately 3 cm diameter and one small piece of skeletal muscle were thawed in a cold chamber on ice; once softened, they were cut to small representative specimens. The tumor sample, upon longitudinal cut, was composed of two types of tissue: an inner core, with calcified matter suggesting residual bone, and a stereotyped white matter that prevailed at the sample periphery. The specimens (24 in total) were then divided into three groups: muscle (A1–A8), oily tumor (B1–B8), and calcified tumor (C1–C8). Scalpel-cut pieces (~10 mg each) were inserted in standard Kel-F disposable inserts (35 μL internal volume) for 4 mm zirconium oxide Bruker rotors (80 μL internal volume) together with 5–10 μL DSS (4,4-dimethyl-4-silapentane-1-sulfonic acid) in D_2_O necessary for referencing and lock routine, sealed and stored at −80 °C. We also generated an average sample (termed “control”) combining specimens from group A (muscle sample expected to be rich in hydrophilic metabolites) and B (tumor area expected to be rich in lipids). To this end, we mixed samples A4 and B4 in one insert. The total number of inserts analyzed was thus 23. Kel-F disposable inserts have a low proton background and higher chemical and biological inertness, and are equipped with a taper and a screw cap ensuring tissue isolation and preservation.

### 4.3. ^1^H HR-MAS NMR

^1^H HR-MAS NMR spectra were acquired on an FT-NMR AvanceTM 500 (Bruker BioSpin GmbH, Rheinstetten, Germany) with a superconducting ultrashield magnet of 11.7 tesla (^1^H frequency: 500.13 MHz) located in the Big Instrument Center (C.I.G.A.), of the University of Milano. HR-MAS NMR spectra were acquired using a sample spinning rate of 4 kHz. Spectra were acquired at 4 °C (± 0.1 °C) to preserve sample integrity and minimize tissue degradation. The temperature was under control of the BVT 3000 Unit (Bruker BioSpin GmbH, Rheinstetten, Germany). The temperature control was calibrated with an internal standard considering the real rotor internal temperature, at the same MAS speed. ^1^H NMR data were acquired using two different pulse sequences (Supplementary Figure S1B): (i) the 1D pulse sequence version of NOESY, with double solvent presaturation during relaxation delay and during mixing time (noesypr1d), with an effective capability of residual tissue water signal elimination, with low influence to vicinal metabolites signals, thus allowing minimum spectrum artefacts and good resolution; (ii) T2-filtered Carr–Purcell–Meiboom–Gill (CPMG) pulse sequence acquisition [90 − (δ − 180 − δ)]_n_, to consistently reduce the line-broadening effects deriving from short T2 components, with standard water presaturation (cpmgpr1d). The two sequences yielded similar spectral resolution. As expected, the CPMG spectra were characterized by reduced peak intensities. However, peak positions, binning regions, and peak behavior along the series of CPMG spectra (group A, B, C) did not change with respect to the NOESY spectra, thus yielding PCA score plots comparable to the ones obtained from the NOESY spectra. Further statistical analyses were therefore performed on the NOESY datasets. All spectra were acquired with the following parameters: 90° pulse of ~20.5 μs (optimized on different set of samples), spectral width of 5.5 kHz (corresponding to 11 ppm), acquisition size and time of 32 K and 3 s, respectively. The relaxation delay was set to 5 s; the number of scans was 120, the mixing time in the NOESY experiment was 60 ms. In the case of cpmg1d pulse sequence, additional parameters were: interpulse delay δ = 1 ms, repetition value L2 = 20, resulting in a total filter time of 40 ms (2δ × L2). Insert corresponding to sample C2 failed to fit into the rotor due to insert deformation, and hence was not analyzed by NMR.

### 4.4. Binning and Profiling

NOESY and CPMG spectra series were processed with MestReNova software version 8.0.2 (Mestrelab Research S.L., Santiago de Compostela, Spain). Spectra were imported, visualized in stacked mode, and automatically phase-corrected with global and metabonomics algorithms. Stacked spectra were apodized along t1 with +1 Hz line broadening and 1 Hz Gaussian bell. Baselines were corrected using fully automatic Whittaker Smoother method; chemical shifts were calibrated on the alanine doublet at 1.46 ppm. In the stacked mode visualization 23 integrals were defined in the spectral region between 0 and 4.5 ppm. Variable binning was applied recursively over all stacked spectra and absolute integrals were output in a matrix with a suitable format for normalization and statistical analysis. Both NOESY and CPMG matrices were composed by 23 variables (bins) and 22 rows (i.e., spectra acquired on 21 samples and 1 control sample composed by mixed muscle-tumor specimen A4/B4). On the basis of already published chemical shift assignments in ^1^H HR-MAS NMR [7,24,25,26,27] we have associated each variable binning region to the metabolites contributing to the integral (Supplementary Table S1).

### 4.5. Statistical Analyses of NMR Data

#### 4.5.1. Univariate Analysis

Peak integrals deriving from NOESY spectra were normalized to the sum of all interval regions for each spectrum and Pareto-scaled. Descriptive statistics, one-way ANOVA with Tukey’s multiple comparison test, linear correlation and regression tests were performed using R statistical suite (http://www.r-project.org/). Mixed muscle tumor specimens (A4/B4) were excluded from the univariate analysis.

#### 4.5.2. Multivariate Analysis

Multivariate statistical analysis through principal component analysis (PCA) was performed using Simca 13.0 software (Umetrics, Umea, Sweden). PCA was applied on the data matrix described above (23 variables and 22 samples). Negative integrals deriving from spectral regions with imperfect baseline correction were changed to 0 values. Normalization was achieved by dividing each integral region by the sum of all interval regions for each spectrum. The normalized integrals were analyzed by PCA after *Pareto*-scaling of the data. PCA analysis included also the mixed muscle tumor specimen (A4/B4).

### 4.6. Histology and Histomorphometry

Samples deriving from ^1^H HR-MAS analysis were recovered from the rotors and embedded in formalin for subsequent histological analysis. Briefly, formalin-fixed samples were rinsed with sodium cacodylate (0.02% and 0.01% overnight), EDTA 0.14% 30 min, and neutral-buffered formalin 10% for 2 days, embedded in paraffin, cut to 5 μm sections with a microtome (Leica 3200, Leica Microsystems GmbH, Wetzlar, Germany) with free floating technique and polylisinate glass (Menzel Glaser, Braunschweig, Germany). After rehydration in warm water, sections were dried on a hot plate, washed with alcohol series (xylene 100%, xylene 50% in ethanol (ETOH), ETOH 100%, ETOH 95%, ETOH 85%, ETOH 70%) and water, stained with hematoxylin and eosin Y (Bio-Optica, Milano, Italy), dehydrated with an alcohol series (ETOH 70%, ETOH 85%, ETOH 95%, ETOH 100%, xylene 50% in ETOH, xylene 100%) and mounted on coverslips with a DPX mounting medium. Images were taken with Axioplan2, Leica Microsystem (Alembic, San Raffaele Scientific Institute, Milano, Italy). Nuclei and adipocytes were counted manually and areas of interest measured with ImageJ software (developed at the National Institutes of Health (Bethesda, MA, USA) available at http://rsb.info.nih.gov/ij/), then normalized by total section area (in pixels). For grouped analyses (ANOVA, charts), histomorphometric data were normalized to pixel area and scaled to range. The mixed muscle-tumor specimen (A4/B4) was excluded from the analysis. Descriptive statistics, one-way ANOVA with Tukey’s multiple comparison test, linear correlation, and regression tests were performed using R statistical suite (http://www.r-project.org/).

### 4.7. Correlation Analyses

Normalization and correlation analysis between NMR features and histomorphometry data were performed using the MetaboAnalyst tool suite (http://www.metaboanalyst.ca) [35]. For metabolites, raw data deriving from the NOESY spectra were normalized by normalizing to the sum of all interval regions and *Pareto*-scaling. Histomorphometry data were independently normalized to area and scaled to range. Normalized data from the two methods were then merged in one matrix set, and then analyzed with Spearman rank-order correlation. We used only information that was confirmed by NMR and histological classification, and excluded outliers (C8, B8), mixed muscle-tumor specimen (A4/B4), as well as misclassified samples C3 and A6 (see Results).

## Supplementary Materials

Supplementary materials can be found at www.mdpi.com/1422-0067/17/11/1814/s1.
