# Supplementary material for: Assessing Heterogeneity of Osteolytic Lesions in Multiple Myeloma by 1H HR-MAS NMR Metabolomics
Source: Int J Mol Sci. 2016 Oct 31;17(11):1814. doi: 10.3390/ijms17111814 (PMC5133815; doi:10.3390/ijms17111814)
Supplement: Supplementary file 1 [file ijms-17-01814-s001.pdf]

Int. J. Mol. Sci. **2016**, *17*, 1814; doi:10.3390/ijms17111814

Supplementary Materials: Assessing Heterogeneity of Osteolytic Lesions in Multiple Myeloma by ^{1}H HR-MAS NMR Metabolomics

Laurette Tavel, Francesca Fontana, Jose Manuel Garcia Manteiga, Silvia Mari, Elisabetta Mariani, Enrico Caneva, Roberto Sitia, Francesco Camnasio, Magda Marcatti, Simone Cenci and Giovanna Musco

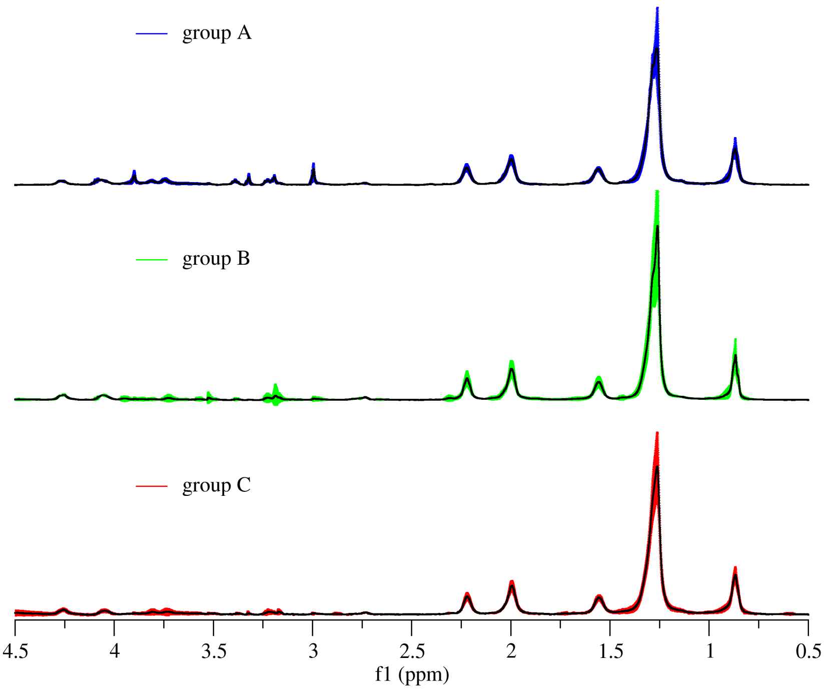

(A)

Figure S1. *Cont.*

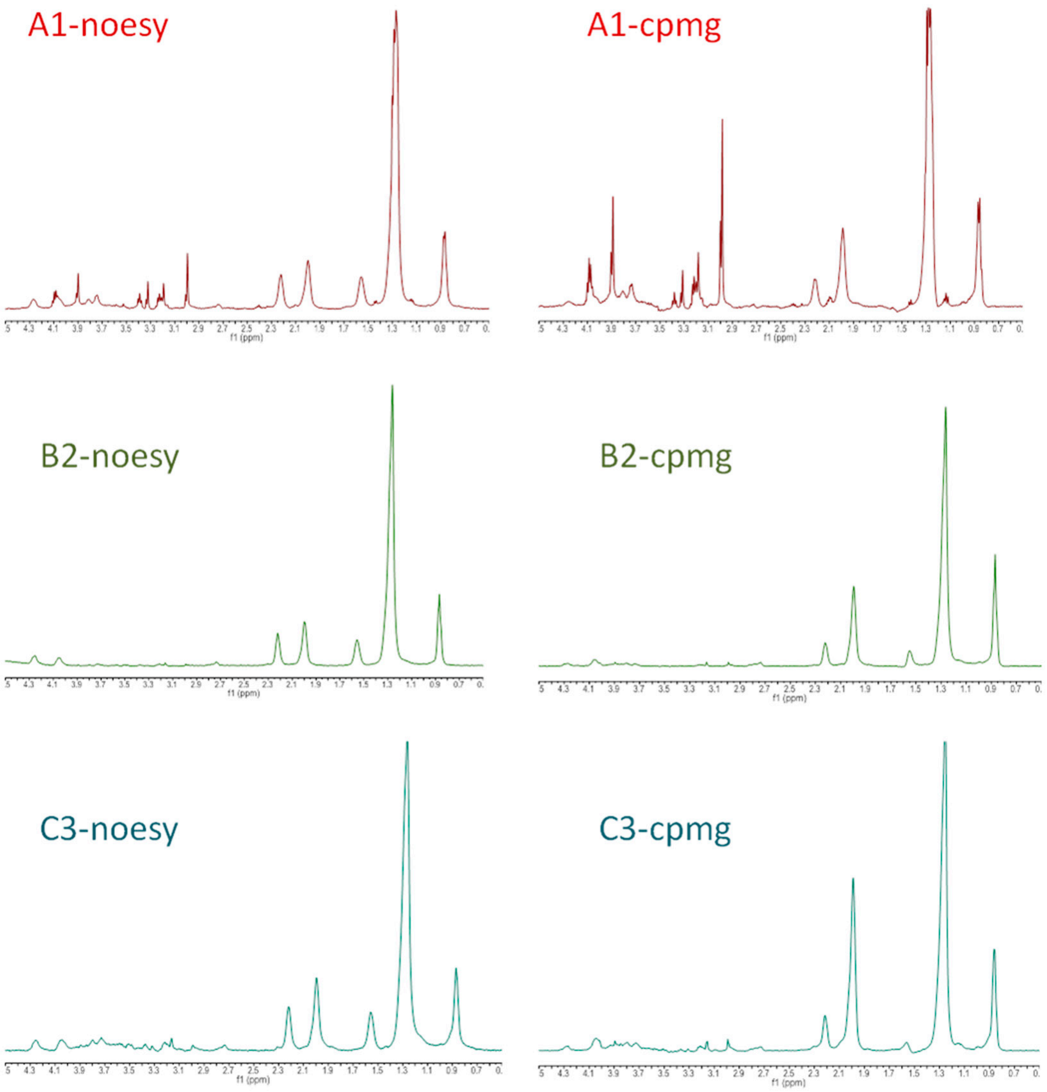

(**B**)

Figure S1. ^{1}H HR-MAS spectra of the 3 different specimens. (**A**) The averaged NOESY spectrum with the corresponding standard deviation calculated for each spectral data point is illustrated for groups A, B and C, respectively; (**B**) Comparison between NOESY and CPMG spectra. One representative sample is illustrated respectively for groups A, B and C. As expected, in the CPMG pulse sequence the relative peaks intensities vary as compared to the NOESY spectrum. However, peak positions, binning regions, and peak behavior along the series of spectra (group A, B, C) do not change. Accordingly, PCA of the spectra acquired with the two sequences are identical.

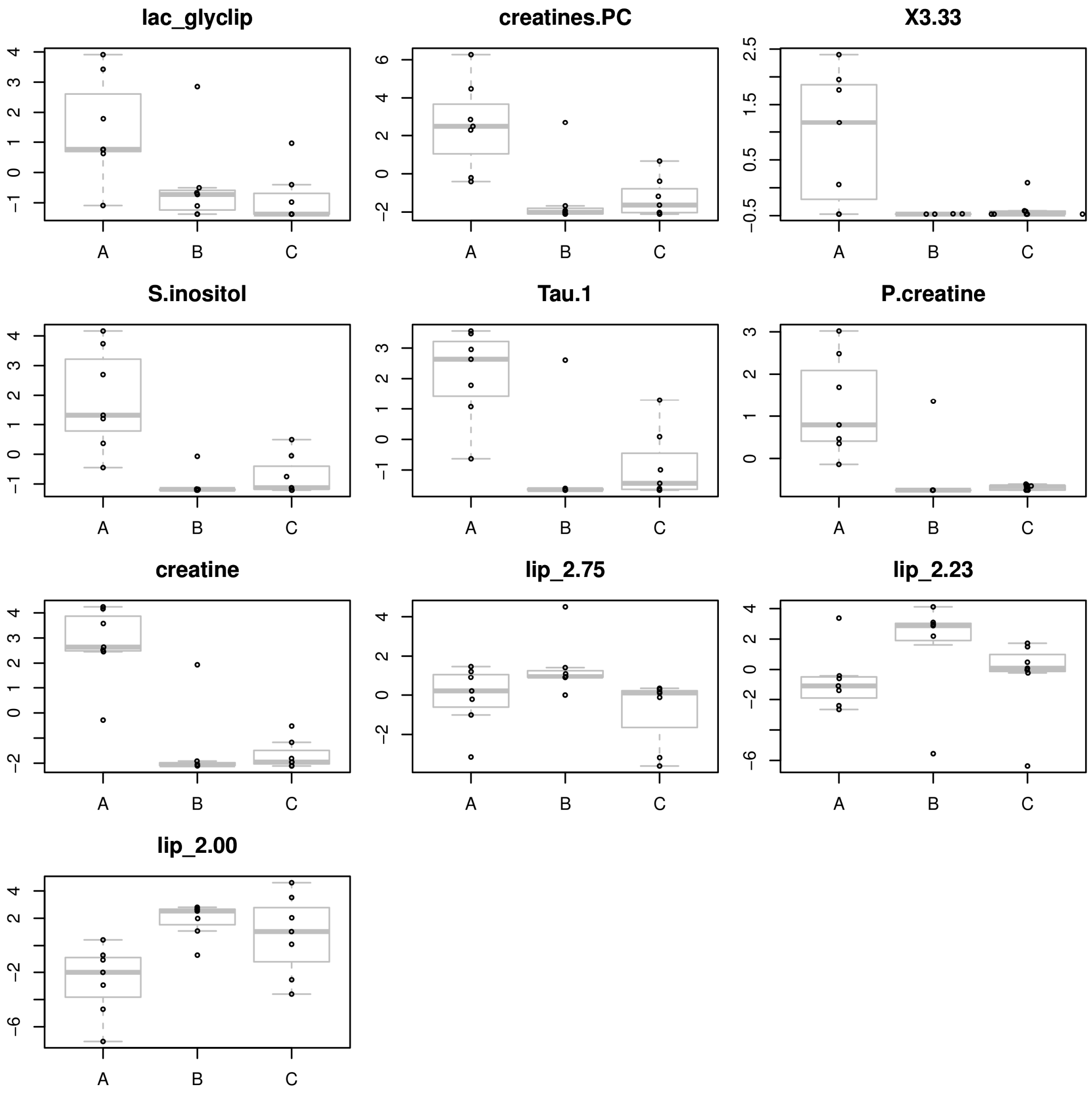

Figure S2. Univariate statistical analysis of ^{1}H HR-MAS NMR metabolic profiles. Normalized and scaled peak integrals of NMR buckets resulting in significant ANOVA *p* values ($p < 0.05$) are represented using Box-Whisker plots as in Figure 2. Lac_glyclip: lactate and glycerolipids; Creatines.PC: creatines and phosphocholines; X3.33: unknown metabolite at 3.33 ppm; S.inositol: Scyllo-inositol; Tau.1: Taurine; P.creatine: phospho-creatine; lip_2.75: lipid at 2.75 ppm; lip_2.23: lipid at 2.23 ppm; lip_2.00: lipid at 2.00 ppm.

Table S1. List and description of variable binning regions used for analysis.

Bin	Spectral Low Field Limit (ppm)	Spectral High Field Limit (ppm)	Label	Description	ANOVA *p* Value	Tukey Post Test (ns: *p* Value > 0.05)		
						Muscle vs. Tumor Oily	Muscle vs. Tumor Calcified	Tumor Oily vs. Tumor Calcified
1	4.36	4.20	glyclip_4.30	glycerolipids	0.790	ns	ns	ns
2	4.12	4.08	lac_glyclip	lactate, glycerolipids	0.016	0.058	0.018	ns
3	4.08	4.00	glyclip_4.05	glycerolipids	0.165	ns	ns	ns
4	3.92	3.87	creatines-PC	creatine and phosphocreatine	0.001	0.002	0.003	ns
5	3.84	3.67	Gly_Glu	glycogen, glucose	0.329	ns	ns	ns
6	3.53	3.48	Gly	glycine	0.887	ns	ns	ns
7	3.41	3.35	Tau	taurine	0.629	ns	ns	ns
8	3.34	3.32	3.33	unknown	0.003	0.005	0.008	ns
9	3.32	3.29	S-inositol	scyllo-inositol	0.000	0.000	0.001	ns
10	3.26	3.13	Tau	taurine	0.001	0.002	0.003	ns
11	3.20	3.14	cholines	choline, O-phosphocholine, glycero-phosphocholine	0.823	ns	ns	ns
12	3.02	2.99	P-creatine	phosphocreatine	0.001	0.003	0.001	ns
13	2.99	2.98	creatine	creatine	0.000	0.000	0.000	ns
14	2.84	2.69	lip_2.75	$=CH–CH_2–CH=$	0.048	ns	ns	0.042
15	2.45	2.37	Glu-Gln	glutamate, glutamine	0.195	ns	ns	ns
16	2.26	2.15	lip_2.23	$CH_2–CH_2CO$	0.240	0.033	0.080	ns
17	2.13	2.06	Glu_Gln	glutamate, glutamine	0.777	ns	ns	ns
18	2.06	1.91	lip_2.00	$CH=CH–CH_2$	0.008	0.008	0.047	ns
19	1.65	1.48	lip_1.55	$CH_2–CH_2CO$	0.393	ns	ns	ns
20	1.47	1.41	Ala	alanine	0.753	ns	ns	ns
21	1.40	1.18	lip_1.30-lac	$(CH_2)n$, lactate, threonine	0.690	ns	ns	ns
22	1.18	1.11	3-HB	3-hydroxybutyrate	0.728	ns	ns	ns
23	0.98	0.79	lip_0.9	CH_3-$(CH_2)n$	0.094	ns	ns	ns
